# Transcriptional profiles of bovine in vivo pre-implantation development

**DOI:** 10.1186/1471-2164-15-756

**Published:** 2014-09-04

**Authors:** Zongliang Jiang, Jiangwen Sun, Hong Dong, Oscar Luo, Xinbao Zheng, Craig Obergfell, Yong Tang, Jinbo Bi, Rachel O’Neill, Yijun Ruan, Jingbo Chen, Xiuchun (Cindy) Tian

**Affiliations:** Center for Regenerative Biology, Department of Animal Science, University of Connecticut, Storrs, Connecticut USA; Department of Computer Science and Engineering, School of Engineering, University of Connecticut, Storrs, Connecticut USA; Institute of Animal Science, Xinjiang Academy of Animal Science, Urumqi, Xinjiang P.R. China; The Jackson Laboratory for Genomic Medicine, Farmington, Connecticut USA; Department of Molecular and Cell Biology, University of Connecticut, Storrs, Connecticut USA

**Keywords:** Pre-implantation development, Embryonic genome activation, RNA-seq, Stage specific module, Hub genes, Bovine, Human, Mouse

## Abstract

**Background:**

During mammalian pre-implantation embryonic development dramatic and orchestrated changes occur in gene transcription. The identification of the complete changes has not been possible until the development of the Next Generation Sequencing Technology.

**Results:**

Here we report comprehensive transcriptome dynamics of single matured bovine oocytes and pre-implantation embryos developed in vivo. Surprisingly, more than half of the estimated 22,000 bovine genes, 11,488 to 12,729 involved in more than 100 pathways, is expressed in oocytes and early embryos. Despite the similarity in the total numbers of genes expressed across stages, the nature of the expressed genes is dramatically different. A total of 2,845 genes were differentially expressed among different stages, of which the largest change was observed between the 4- and 8-cell stages, demonstrating that the bovine embryonic genome is activated at this transition. Additionally, 774 genes were identified as only expressed/highly enriched in particular stages of development, suggesting their stage-specific roles in embryogenesis. Using weighted gene co-expression network analysis, we found 12 stage-specific modules of co-expressed genes that can be used to represent the corresponding stage of development. Furthermore, we identified conserved key members (or hub genes) of the bovine expressed gene networks. Their vast association with other embryonic genes suggests that they may have important regulatory roles in embryo development; yet, the majority of the hub genes are relatively unknown/under-studied in embryos. We also conducted the first comparison of embryonic expression profiles across three mammalian species, human, mouse and bovine, for which RNA-seq data are available. We found that the three species share more maternally deposited genes than embryonic genome activated genes. More importantly, there are more similarities in embryonic transcriptomes between bovine and humans than between humans and mice, demonstrating that bovine embryos are better models for human embryonic development.

**Conclusions:**

This study provides a comprehensive examination of gene activities in bovine embryos and identified little-known potential master regulators of pre-implantation development.

**Electronic supplementary material:**

The online version of this article (doi:10.1186/1471-2164-15-756) contains supplementary material, which is available to authorized users.

## Background

Mammalian pre-implantation embryonic development is a complex process including fertilization, cleavage divisions, compaction, and blastulation. During this process, massive degradation of oocyte-stored maternal RNA/proteins and gradual activation of the embryonic genome take place [1]. Earlier studies employing RNA polymerase II inhibitor suggested that the timing of EGA is correlated with the speed of embryonic development. For example, α-amanitin halted embryo development at the 2-cell stage in mice [2–4] and between 4- and 8-cell stages in humans [5]. However, the exact timing of EGA in bovine is still debated. Developmental block of cultured bovine embryos occur between the 8- and 16-cell stages [6–8], suggesting EGA at this transition. Similar conclusion was reached by studying expression profiles of bovine in vitro embryos using microarray [9] or the RNA sequencing (RNA-seq) technology [10]. However, a microarray study utilizing pooled in vivo bovine embryos suggested that bovine EGA occurs between the 4- and 8-cell stages [11].

To date, the most comprehensive transcriptome profiling of bovine in vivo embryos was carried out using the Affymetrix GeneChip Bovine Genome Array. Although this microarray contains roughly 23,000 bovine transcripts, these represent only 12,752 genes (personal communications with Affymetrix), approximately half of the mammalian genes. Previous data are also impacted by hybridization variations of microarray and the use of pooled embryos [8, 11]. Although more comprehensive data were obtained using RNA-seq, they were restricted to bovine blastocysts only [12, 13] or with the use of in vitro embryos [10]. As a result, the complete descriptions of gene activities during bovine in vivo embryonic development are still not available. Moreover, the timing of EGA should not be established using expression data from half of the genome or in vitro embryos. The RNA-seq technology provides unique benefits for studying gene expression with high resolutions and reproducibility, as well as for detecting novel transcripts and alternative splicing events [14, 15]. Here we applied the Solid RNA-seq platform on single in vivo matured oocytes and in vivo developed embryos from the 2-cell to the blastocyst stages and obtained their comprehensive transcriptome dynamics. The identification of highly connected, yet relatively little known or completely unknown hub genes that are potentially master regulators of gene expression opens up unprecedented opportunities for further understanding of early development. Furthermore, we used RNA-seq datasets recently generated in humans and mice and carried out a comprehensive stage-specific comparison across the three mammalian species. We found that the three species shared more maternally deposited genes than embryonic genome activated genes. More significantly, there are more similarities between bovine and human embryonic transcriptomes than those between humans and mice. The data obtained here will function as a comprehensive reference base for embryos generated from reproductive biotechnologies in the bovine as well as in the human for which the use of in vivo embryos is highly limited.

## Results

### Expression profiles of bovine in vivo matured oocytes and pre-implantation embryos

**Figure 1:**
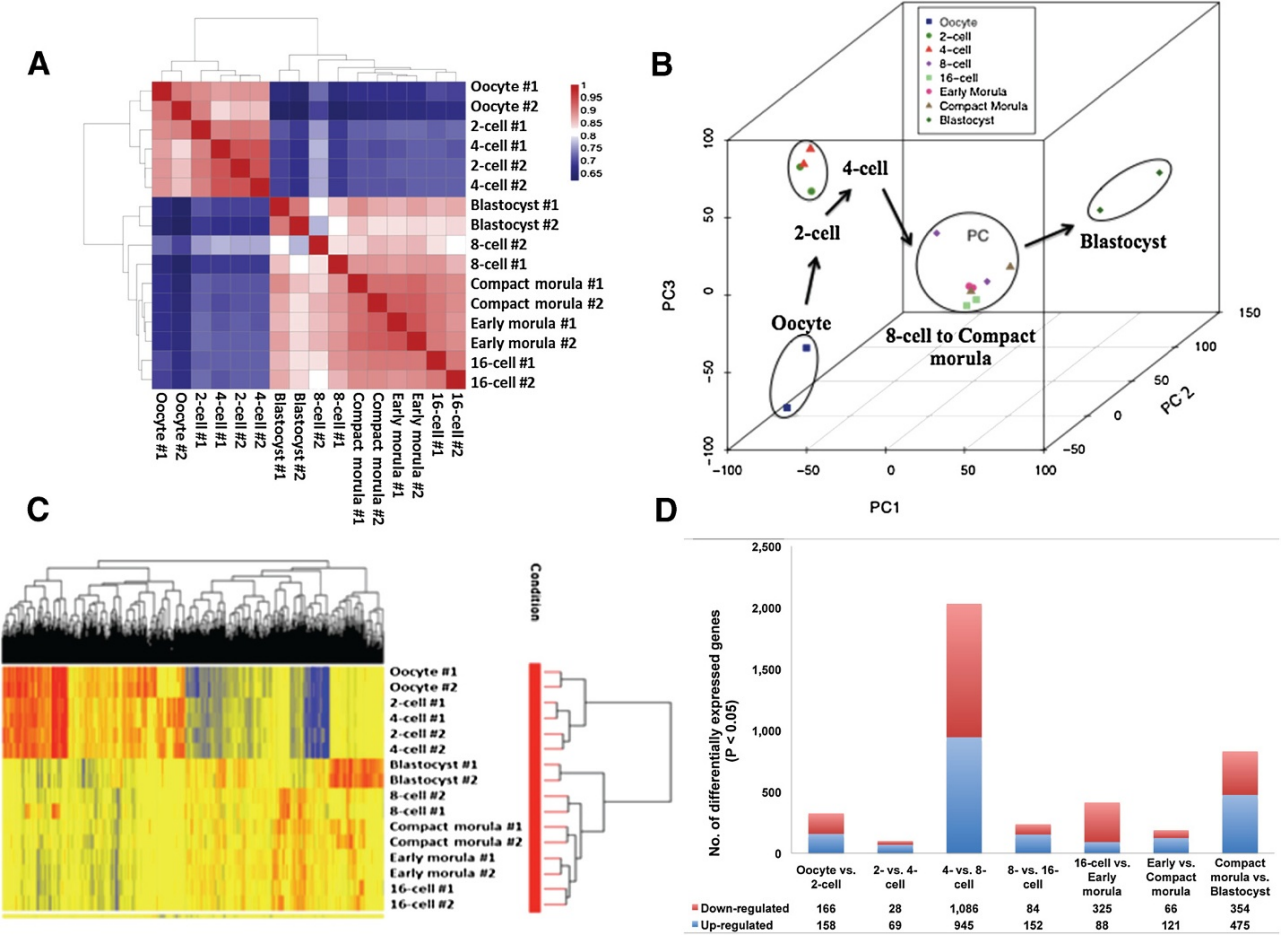
**Correlation and hierarchical analyses of transcriptomes of bovine in vivo developed oocytes and embryos. (A)**. Heatmap of duplicate samples of the same stages of bovine embryonic development. The color spectrum, ranging from red through white to blue, represents Pearson correlation coefficients ranging from 1 to 0.53, indicating high to low correlations. All duplicate samples are highly correlated in Pearson coefficients demonstrating the reproducibility of the procedures. **(B)**. Principal component analysis (PCA) of the transcriptomes for seven stages of in vivo developed bovine embryos and oocytes. Embryos from the same stage are shown by symbols of the same shape. The arrows indicate the developmental direction of the embryos. PC1, PC2 and PC3 represent the top three dimensions of the differentially expressed genes among the preimplantation embryos. **(C)**. Hierarchical clustering of differentially expressed genes in in vivo developed bovine oocytes and embryos. Two major clusters are shown, one consisted of the matured oocytes and embryos at the 2- and 4-cell stages. The second cluster is consisted of embryos at the 8-, 16-cell, early morula, compact morula and blastocyst stages. The clear separation of embryos into two groups demonstrated the timing of EGA in cattle at the 4- to 8-cell transition. The color spectrum, ranging from red through yellow to blue, indicates normalized levels of gene expression. **(D)**. The numbers of differentially expressed genes in consecutive stages of bovine in vivo pre-implantation development (P < 0.05).

Using linearly amplified RNA from single oocytes/embryos, we obtained approximately 430 million sequencing reads from duplicate samples of bovine in vivo pre-implantation embryos at 8 stages of development (Additional file 1: Table S1). The raw FASTQ files and normalized read counts per gene are available at Gene Expression Omnibus (GEO) (http://www.ncbi.nlm.nih.gov/geo) under the accession number GSE59186. High Pearson correlation coefficients were found among biological replicates of the same developmental stage (Figure 1A, Additional file 2: Table S2), demonstrating the reproducibility of sample preparation and the sequencing technology. Instead of hundreds of expressed genes reported earlier [8, 11], the total numbers of detectable genes ranged from 11,488 to 12,729 from oocytes to blastocysts in our study with the use of RNA-seq (Table 1 and Additional file 3: Table S3). For the first time, it is revealed that the bovine oocytes and early embryos expressed roughly 50% of the total estimated 22,000 genes in the bovine genome.

**Table 1:** **The numbers of genes detected in bovine in vivo matured oocytes and each stage of in vivo embryonic development**

Stage	No. of genes (FPKM > 0.1)
**Oocyte**	11488
**2-cell**	12678
**4-cell**	12718
**8-cell**	12729
**16-cell**	11973
**Early morula**	11670
**Compact morula**	11910
**Blastocyst**	11924

FPKM: fragments per kilobase of exon per million fragments mapped.

Pearson correlation coefficients and principal component analyses (PCA) of all detected genes revealed two distinct segmentations of bovine pre-implantation development: the first from the oocyte to the 4-cell stage; and the second from the 8-cell to the blastocyst stage (Figure 1A, B). This segmentation demonstrated that EGA in bovine occurs between the 4- and 8-cell stages. This timing concurred with the conclusion by Kues et al. [11] yet contrasted with those of all other studies [6–8, 10]. Two additional segmentations were also worth-noting: the first from oocyte to 2-/4-cell stages, and the second from 8-cell/morula to the blastocyst stage (Figure 1B). These divisions were likely results of dramatic degradation of maternal RNAs and early differentiation, respectively.

### Differentially expressed genes during bovine in vivo pre-implantation development

Although the total numbers of genes expressed by embryos of different stages did not vary much, the actual genes expressed during early development are dramatically different. A total of 2,845 unique genes were identified to be differentially expressed between all consecutive stages of development (P < 0.05). Similar to Pearson correlations on all detected genes, hierarchal clustering of the differentially expressed genes also partitioned pre-implantation development into two distinct clusters (Figure 1C), that from oocytes to 4-cell and from 8-cell to blastocysts, which confirms the timing of EGA. The majority of the differentially expressed genes, 2,031, were found between the 4- to 8-cell stages, providing another confirmation that bovine EGA occurs at this transition. Among these genes, 1,086 and 945 were down- and up-regulated, respectively (Figure 1D). The down-regulated genes are involved in reproduction, transcription and cell cycle regulations. Conversely, the up-regulated genes, representing those transcribed from the embryonic genome, are involved in translation, ATP metabolic process as well as RNA processing (Additional file 4: Table S4). The second largest change in gene expression occurred from compact morula to blastocyst, with 829 genes differentially expressed (Figure 1D), suggesting that specific genes were necessary during the blastulation and early differentiation processes. The biological processes significantly represented at this transition included cell proliferation, transport and early differentiation (Additional file 5: Table S5). This burst of protein production and cell division may be necessary to prepare the blastocyst for the upcoming coordinated differentiation.

Two minor EGA events were also identified in addition to the major EGA between the 4- and 8-cell stages: one from the oocyte to the 2-cell stage and the other from the 16-cell to the early morula stage, with 324 and 413 genes differentially expressed, respectively (Figure 1D). Between the oocytes and the 2-cell embryos, 166 of the 324 differentially expressed genes were down-regulated. These represent rapid degradation of the maternally stored transcripts. Gene ontology analysis indicated significant over-representation of elements involved in cell cycle and mitosis II (Additional file 6: Table S6), suggesting that the 2-cell embryos reprogrammed its cell cycle regulation from that of an arrested state to an active mode of cell division through changes of gene expression. The second minor EGA, between the 16-cell embryo and early morula, included 413 differentially expressed genes (Figure 1D). These genes may play important roles during the development of tight junctions and other processes of compaction. Interestingly at this transition, we found a high enrichment of genes involved in stem cell maintenance and development, suggesting that genes for pluripotency are active long before the formation of the inner cell mass (Additional file 7: Table S7).

In spite of the aforementioned differences, there are also wide-spread similarities in the expression profiles between the 2- and 4-cell embryos, the 8- and 16-cell embryos, as well as the early and compact morulae (Figure 1D). Specifically, only 97 genes were differentially expressed between the 2- and 4-cell embryos (Additional file 8: Table S8). Moreover, among the more than 11,000 genes commonly expressed by both the 8- and 16-cell embryos, only 236 genes were differentially expressed (Additional file 9: Table S9). Likewise, as few as 187 differentially expressed genes were found among the 10,843 commonly expressed genes between the early and compact morulae (Additional file 10: Table S10).

To confirm the throughput results from RNA-seq, we performed quantitative real-time PCR (qRT-PCR) on 10 genes using in vivo embryos at the 4- and 8-cell stages (n = 3). Among the selected genes, five (*GATA6, GNB2L1, BAD, H2AFZ* and *NANOG*) were up-regulated and five (*GDP9*, *DNMT1*, *ZP2*, *STAT3* and *OOER*) were down-regulated between these two stages. The qRT-PCR results substantiated those from RNA-seq (Table 2).

**Table 2:** **Quantitative real-time RT-PCR (qRT-PCR) results of 10 selected genes between 4- and 8-cell stage embryos**

Comparison	Gene symbol	Log (fold change) RNA-Seq	Expression	Log (fold change)* qRT-PCR
**4- vs. 8-cell embryos**	*GATA6*	8.4	Up	10.6
	*GNB2L1*	8.2	Up	9.2
	*BAD*	6.4	Up	8.4
	*H2AFZ*	8.2	Up	10.3
	*NANOG*	11.5	Up	7.8
	*GDP9*	-3.8	Down	-2.1
	*DNMT1*	-3.2	Down	-2.6
	*ZP2*	-3.5	Down	-4.4
	*STAT3*	-2.8	Down	-2.3
	*OOER*	-2.7	Down	-2.0

*Fold change is expressed as the ratios of the values of the 4-cell embryos (n = 3) divided by those of the 8-cell embryos (n = 3). Real time RT-PCR results substantiated the differential gene expression patterns from RNA-seq.

### Cluster profiles of differentially expressed genes

**Figure 2:**
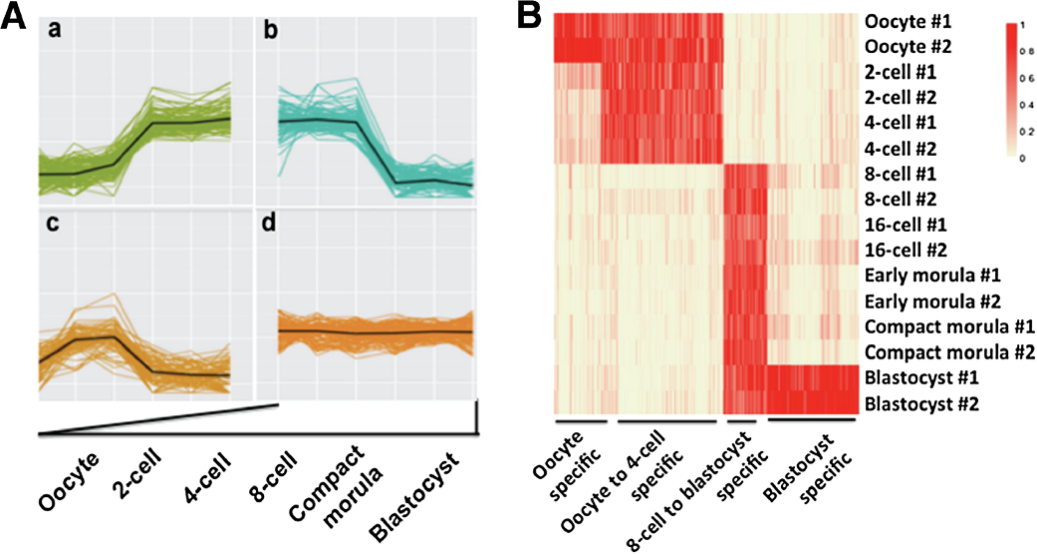
**Distinct patterns and dynamics of gene expression during bovine in vivo pre-implantation development. (A)**. Representative clusters of expression dynamics during early development. Genes were clustered to be increased (a), decreased (b), increased first and then decreased (c), and maintained relatively constant levels of expression (d). **(B)**. Identification of stage-specific/enriched genes by cluster analysis. Groups of genes were found to be only expressed in oocytes and blastocysts, enriched in oocytes to 4-cell embryos, and 8-cell to blastocysts. The color spectrum, ranging from red to white, represents high to low levels of gene expression.

Although as many as 2,845 differentially expressed genes were identified, the pattern of their dynamic changes can be categorized into as few as 30 distinct clusters , labeled as Clusters 1 to 30 (Additional file 11: Figure S1; Additional file 12: Table S11). These clusters can be further assigned to four main groups of different dynamic patterns (Figure 2A). The first group, including Clusters 1, 7, 9, 11, 13, 14, 15, 17, 19, 22, 24, 27 and 29, represents genes that increased their expression levels during pre-implantation. All clusters in this group, with the exception of Clusters 11, 17 and 29 showed a dramatic increase at the 8-cell stage, indicating that they are transcribed from the embryonic genome. Genes in these clusters include developmentally important ones such as *GATA6*, *H2AFZ* and *NANOG.* Interestingly, genes in Clusters 11 and 29, including *GATA3* and *DSP,* peaked at the blastocyst stage, suggesting their roles in blastocyst formation and early differentiation. The second dynamic expression pattern, including Clusters 2, 5, 6, 8, 16, 18, 23, 25, 26 and 28, represents genes that underwent an overall trend of decrease, suggesting continued degradation over the pre-implantation period. Of special interest was a sharp decrease during the 4- to 8- cell transition in Clusters 6, 8 and 28, including oocyte-specific genes such as *ZP2* and *WEE2*, demonstrating the lack of their involvement in embryo development. The decrease of genes in this group may also be a pre-requisite for EGA. The third expression pattern, including Clusters 4, 10, 20 and 21, contains genes that first increased and then decreased their expression levels. Among these, Clusters 4 and 10 were up at the 8-cell stages and then declined, suggesting that these genes, such as *BAD, APOPT1* and *GNAT1*, are only involved in the activation of the embryonic genome. The last group, including Clusters 3, 12 and 30, represents genes that maintained relatively constant levels of expression throughout all stages studied. Members from this group such as *ATP1A1*, *ATP5F1* and *RALB* are involved in ion exchange, energy metabolism and signal transduction, suggesting their necessary roles during the entire process of pre-implantation development. It is possible that some of the transcripts in this group were stable maternal RNAs that were never degraded while others were maintained by the early embryos. Nonetheless, genes in this group are good loading control candidates for gene expression quantifications.

In addition to the dynamic changes of gene expression, we also identified genes that are only enriched in one particular stage of development. Specifically, a group of 119 genes were enriched only in the matured oocytes (Figure 2B, Additional file 13: Table S12). These transcripts were degraded after fertilization and remained suppressed during embryo development. Apart from well-known/studied genes such as *H1FOO*, we identified many less known/not annotated genes such as *LOC782175* and *LOC536606*. These genes likely have limited roles in embryo development but are important in maintaining oocytes at the matured stage. Further investigations into their roles in oocytes will enhance our understanding of the mechanism for meiotic arrest. Another group of 234 genes, including *DNMT3A*, *GATA3*, *CD9* and *APOP1*, were only enriched at the blastocyst stage (Figure 2B, Additional file 13: Table S12). Groups of genes were also found to be enriched in a short duration of development such as 310 during oocyte to 4-cell stage and 111 during 8-cell to blastocyst stage (Figure 2B, Additional file 13: Table S12).

### Stage-specific and cross-species gene expression comparisons

**Figure 3:**
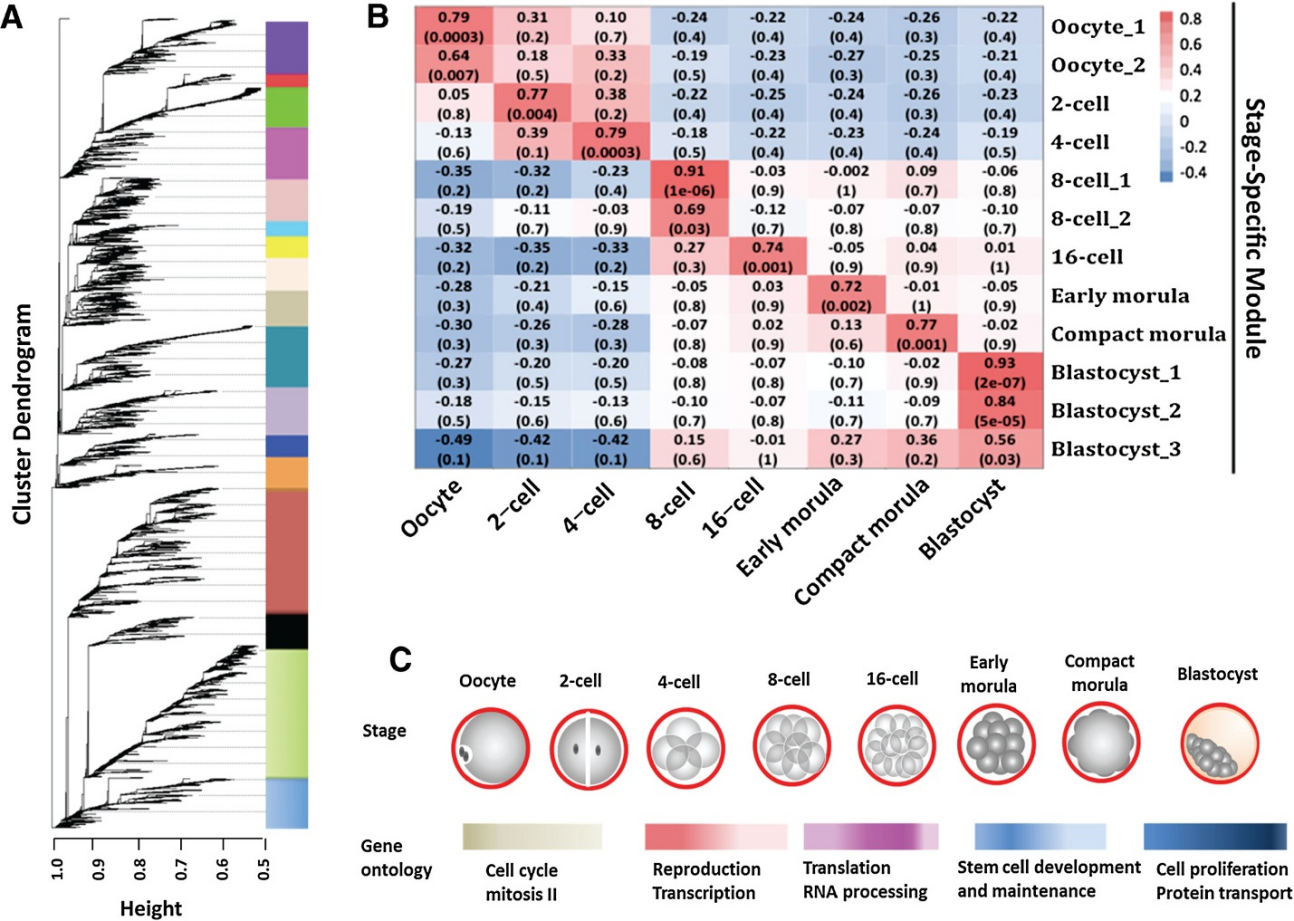
**Co-expression network analysis of bovine pre-implantation development. (A)**. Hierarchical cluster tree showing modules of co-expressed genes identified by WGCNA. A total of 17 co-expressed modules were found during bovine pre-implantation development and were represented by branches and labeled by different colors to the right of the tree. The height (X-axis) indicates levels of correlation. **(B)**. Heatmap of correlations (and corresponding P-values) between co-expressed modules and stage of development. The color scheme, from blue through white to red, indicates the levels of correlation, from low to high. The stage-specific modules identified are highly correlated (i.e. over-expressed) with distinct developmental stages (columns). **(C)**. Functional terms of stage-specific modules of co-expressed genes during bovine pre-implantation development. A systematic and sequential changes in functions of co-expressed genes were observed as the embryos progress through early development.

In addition to analyzing changes of individual genes, we also examined gene-interactions by identifying modules of genes that were co-expressed. Gene co-expression suggests their involvement in a common network of biological processes and functions [16]. Using weighted gene co-expression network analysis (WGCNA) [17, 18] we identified 17 distinct co-expression modules from 13,127 detected genes in our RNA-seq dataset (Figure 3A). Twelve of these modules were stage-specific, i.e., these modules included genes that were overexpressed in a particular embryonic stage (Figure 3B, Additional file 14: Table S13) and can be used to represent the corresponding stage of development. Interestingly, analysis of the functions of genes in these modules revealed a sequential progression of stage-specific core gene networks. It migrated from cell cycle in oocytes, to regulation of transcription in 4-cell embryos, to translation in 8-cell embryos, to stem cell development, maintenance and differentiation in morulae, and finally to cell proliferation and protein transport in blastocysts (Figure 3C). Such coordinated changes of functional pathways are reflective of the little-known developmental programming.

**Figure 4:**
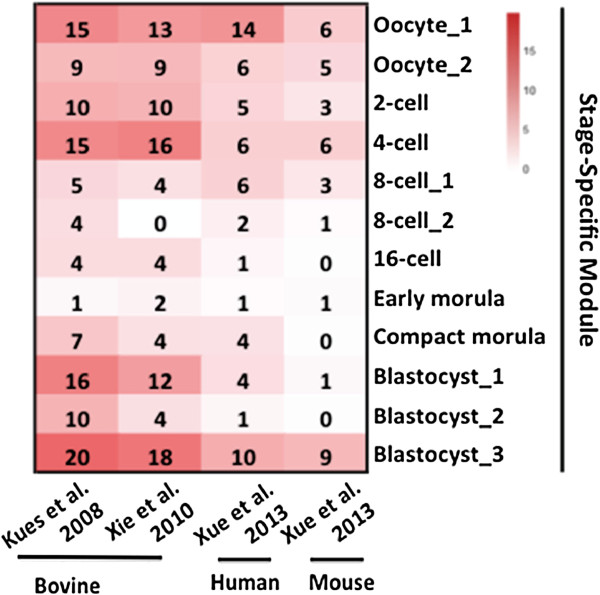
**Heatmap of module preservation of stage-specific gene co-expression among bovine, human and mouse oocytes and embryos.** Commonly expressed orthologs from the present study and those published previously as indicated on the X-axis, including two from bovine microarray studies [9, 11] and an RNA-seq study in humans and mice [19] were identified and included. The labels on the Y-axis are stage-specific modules of co-expressed genes. The color scheme, from white to red, indicates low to high levels of preservation.

To explore the conservation and divergence of genes in the 12 stage-specific co-expression modules within and across species, we downloaded the raw datasets from two previously published microarray studies of the bovine [9, 11] and one recently published RNA-seq study of the human and mouse [19]. We then identified 8,103, 9,648 and 8,705 commonly expressed orthologs from bovine, humans and mice against our expression dataset. The modulePreservation function of WGCNA was used to calculate the Z-statistics [20], which is a measure of the level and pattern of the connectivity of co-expressed genes. As expected, the 12 stage-specific modules were more significantly preserved within species than between species. Specifically, 5 out of the 12 bovine stage-specific modules were strongly (Z ≥ 10) preserved in the two published bovine microarray datasets of similar samples, another 5 were weak to moderately preserved (2 < Z < 10; Figure 4). To date, there is only one published report [19] of cross-species comparisons using co-expression module data from human and mouse embryos. Here we conducted the first study assessing the cross-species preservation using data from the three available mammalian species, bovine, human and mouse. Remarkably, most bovine stage-specific modules were at least moderately preserved with those of the human but less with those of the mouse (Figure 4), suggesting that the human not only share more similarities with the bovine than with the mouse in genome sequences [21, 22] but also in embryonic gene-expression patterns, and thus supporting the notion that bovine embryos are better models for human embryonic development than their mouse counterparts.

**Figure 5:**
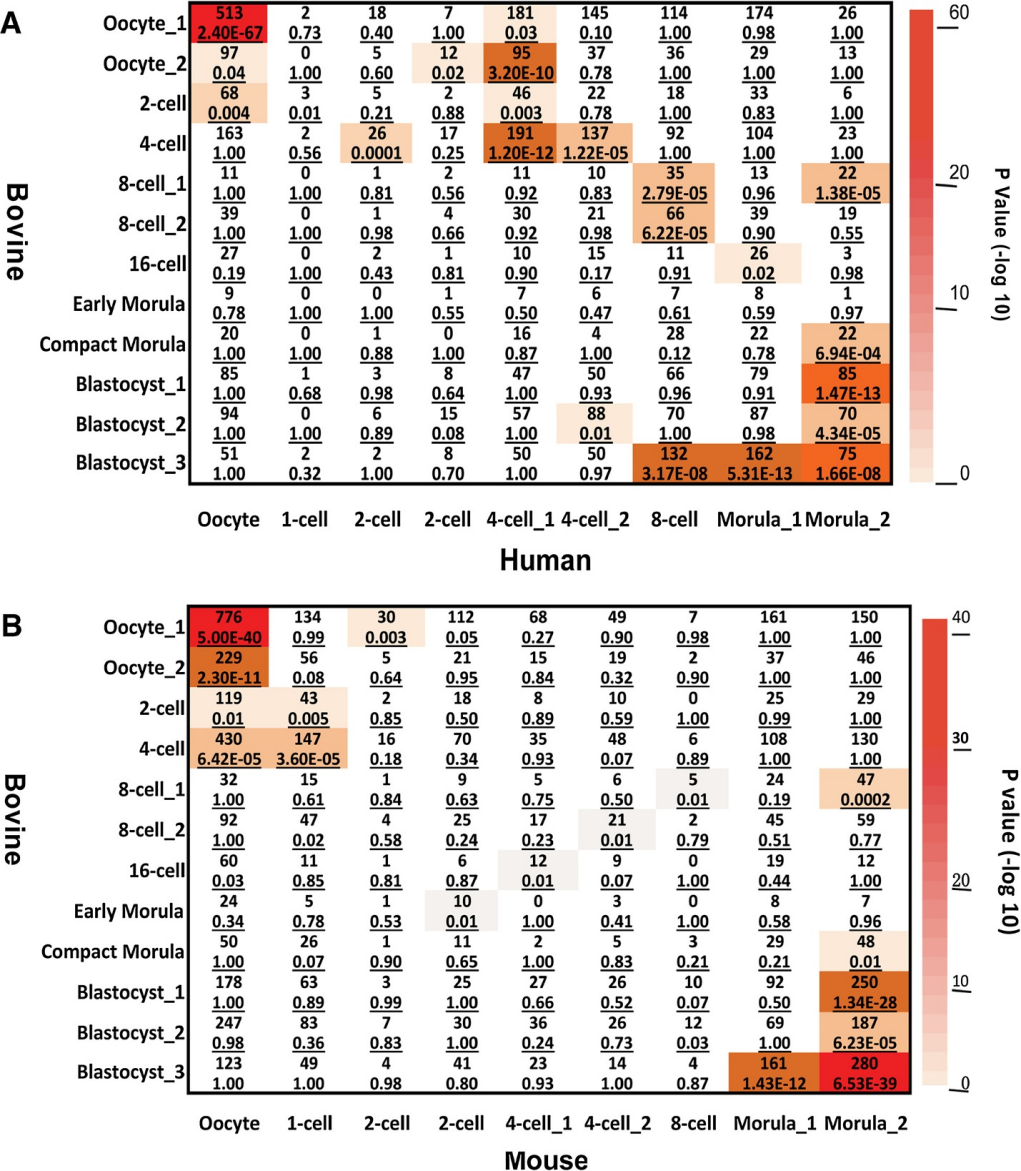
**Divergence of stage-specific gene co-expression among bovine, human and mouse oocytes and embryos. (A)**. Heatmap of gene overlap between independently constructed bovine and human modules. The X- and Y-axes show human (n = 9) and bovine stage-specific modules (n = 12), respectively. Each cell contains the number of intersecting genes and the corresponding P-value (-log10) of the intersection. Significant gene overlaps were found in nearly all stages of development between the human and bovine. **(B)**. Heatmap of gene overlap between independently constructed bovine and mouse modules. The X- and Y-axes show mouse (n = 9) and bovine stage-specific modules (n = 12), respectively. Each cell contains the number of intersecting genes and the corresponding P-value (-log10) of the intersection. Significant gene overlaps were only observed in oocytes and morula/blastocysts between the bovine and mouse.

To further characterize the conservation and variation of functional modules among species, we conducted module analysis using WGCNA on all detected genes, 14,766 and 13,879, respectively, from the RNA-seq datasets of the human and mouse [19]. We then compared the gene lists within the co-expressed modules of the three species. Intriguingly, there are significant overlaps of genes in the bovine and human stage-specific modules (P < 10^-4^; Figure 5A, Additional file 15: Table S14). Of note, many genes in modules specific to the bovine oocyte, 4-cell, 8-cell and morula stages overlapped with those at the corresponding stages in humans. For example, 513 genes (P < 10^-60^) overlapped between bovine and human oocytes alone. These genes are involved in protein transport and cell cycle processes. Similarly, highly significant overlap in module genes were observed during bovine and human late pre-implantation development (from 8-cell to blastocyst) although some bovine stage-specific module genes can be found in multiple stages of human development, and vice versa (Figure 5A). GO analysis of these overlapped module genes indicate significant over-representation of translation, RNA processing, generation of precursor metabolites and energy. To our surprise, as many as 95 genes (P < 10^-9^) in the bovine oocyte-specific module were found in the human 4-cell specific module, giving the apparent suggestion that the progression of embryo development in the bovine may be more rapid than that of humans. This certainly contradicts with the established observations that bovine in vivo embryo development is 8 days (oocytes to blastocysts) [23] while in humans it is 5 days [24]. One possible explanation to this is the diversity of embryonic programming, e.g., human embryos prepare for an invasive type of implantation while bovine embryos only attach to the uterus [25]. Consistent with this possibility, GO analysis showed that these overlapped genes are related to signal transduction such as Ras and small GTPase signaling. Together, these results suggested that bovine and humans share many core transcriptional programming, while differ in stage-specificity and timing. In the contrary, overlaps of genes between bovine and mouse stage-specific modules were only observed before EGA and after morula formation (Figure 5B, Additional file 15: Table S14). For example, genes in the mouse oocyte-specific module overlapped significantly with those specific to bovine oocyte (P < 10^-10^), 2-cell (P < 10^-2^) and 4-cell stages (P < 10^-4^). Meanwhile, genes specific to the mouse morula module significantly overlapped with those in the bovine 8-cell (P < 10^-3^), compact morula (P < 10^-2^) and blastocyst modules (P < 10^-12^). These results showed that mouse early and late pre-implantation genes are spread over a large period of the bovine development, consistent with the speed of embryo development in these two species.

Collectively, our results show that the three mammalian species share more maternally deposited genes than those expressed after EGA. Based on overlapped genes found in modules prior to EGA, bovine maternal detritus (RNA and protein) occurs later than that in the mouse but slightly earlier than that in the human, despite the longer bovine pre-implantation development.

### Identification, visualization and validation of hub genes

**Figure 6:**
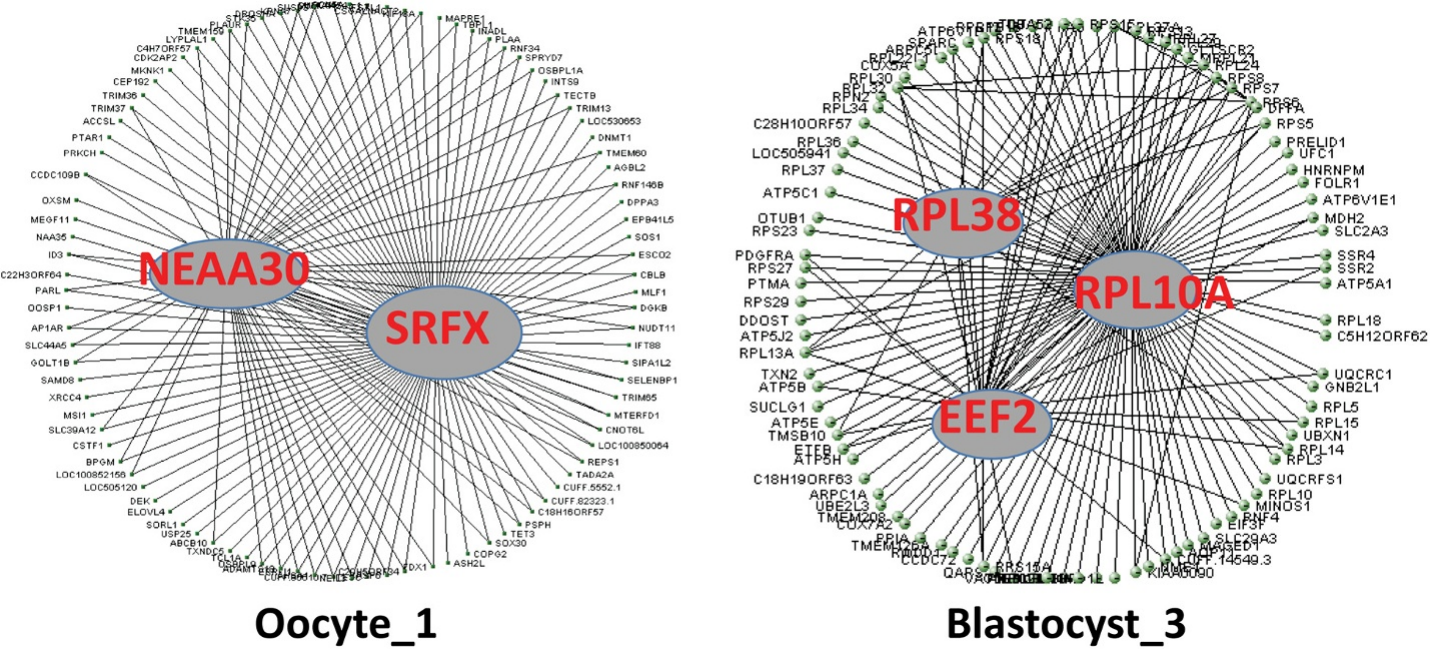
**Representative, highly correlated hub genes in bovine oocytes (** ***NEAA30, SRFX*** **) and blastocysts (** ***RPL38, EEF2, RPL10A*** **).**

In order to identify genes that are central and highly-connected within the stage-specific modules, we conducted hub gene identification analysis. Hub genes are highly correlated within the stage-specific modules and are conceptual and concrete representatives of the corresponding modules. For each stage-specific module, we assigned all genes with Pearson correlation coefficients greater than 0.9 as its hub genes (Table 3). Furthermore, to explore the connections among hub genes, we examined the top 200 connections of the top 150 hub genes (highly correlated hub genes) for each stage-specific module and visualized them in VisANT (Table 4, Figure 6). The full lists of these genes can be found in Additional file 16: Table S15. Although there are well-studied genes such as *RALB* in oocytes and *DNMT3A* in blastocysts, many of these genes are surprisingly either under-studied in embryonic development or un-annotated, and are thus less known/unknown in this process. For example, *LOC100137763* and *LOC100849216* were highly correlated, un-annotated hub genes in bovine mature oocytes and blastocysts, respectively (Table 4, Additional file 17: Figure S2). The highly correlated hub genes reported here are likely key players for their specific stage(s) of development and may function as “master regulators” of gene expression and stage transition in early development. Further investigation into their identities and functions will greatly enhance our understanding of embryo development and our ability to manipulate embryos through biotechnologies.

**Table 3:** **The numbers and validation results of hub genes in bovine in vivo oocytes and embryos**

Stage-specific modules	Total no. of genes/module	Total no. of hub genes/module	No. (%) hub genes (validated in at least one dataset)*	No. (%) hub genes (validated in Kues et al. 2008 [ [11]])
**Oocyte_1**	2347	650	211 (32%)	117 (18%)
**Oocyte_2**	815	125	24 (19%)	6 (5%)
**2-cell**	444	112	12 (11%)	11 (10%)
**4-cell**	1868	299	54 (18%)	19 (6%)
**8-cell_1**	229	52	7 (13%)	5 (10%)
**8-cell_2**	640	172	14 (8%)	13 (8%)
**16-cell**	247	27	1 (4%)	0 (0)
**Early morula**	120	15	2 (13%)	2 (13%)
**Compact morula**	311	41	7 (17%)	7 (17%)
**Blastocyst_1**	1049	274	132 (48%)	116 (42%)
**Blastocyst_2**	1366	366	59 (16%)	44 (12%)
**Blastocyst_3**	1010	118	65 (55%)	44 (37%)

More than one modules were found for oocytes, 8-cell embryos and blastocysts.

*two bovine microarray [9, 11], one human and one mouse RNA-seq datasets [19] were used.

**Table 4:** **Highly correlated hub genes in bovine stage-specific modules**

Stage-specific module	Hub genes
**Oocyte_1**	*SRPX, NAA30*
**Oocyte_2**	*LOC100137763, PAX3, RALB, SMC1B, UNC13C, VANGL1*
**2-cell**	*CAPRIN2, LACC1, LOC616167, NLRP9, ZGLP1, POL*
**4-cell**	*CNTNAP2, TPM3*
**8-cell**	*LOC519952, LOC789391, LYSMD3, TBXAS1, THAP8*
**16-cell**	*ARL10, FAM84B, LOC790411, CCDC39*
**Early morula**	*LGALS9, STAC, LOC100140626*
**Compact morula**	*APOBR, GALNTL1, LRP8, PCDH10, RGS20, HOXA11, LOC781048*
**Blastocyst_1**	*DNMT3A, ATP6V0A4, FAM115C, LGALS1, SLC9A3R1*
**Blastocyst_2**	*BCAM, BPIFA1, LOC100849216, PLXNA3, SHROOM2, SLC16A7*
**Blastocyst_3**	*EEF2, RPL10A, RPL38*

Multiple modules exist for oocytes and blastocysts.

Cross-species analysis showed higher degrees of hub gene validation at the oocyte and blastocyst stages than at other stages. For example, 32% (211 genes) and 48% (132 genes) of all hub genes in the oocyte_1 and blastocyst_1 modules, respectively, were validated in at least one dataset (Table 3, Additional file 16: Table S15). Fewer hub genes from the 2-cell to morula stage were successfully validated among species. For example, only 4% and 8% of hub genes were validated at the 16-cell and 8-cell stages, respectively. This low degree of validation reflected the divergence in the stage-specificity and timing of transcriptional programming. Unexpectedly, we observed relatively low number/percentage of validated hub genes against the two previously published bovine datasets [9, 11] (Table 3), likely because of the low coverage of microarray and/or the relatively low resolution of microarray data.

### Pathways in stage-specific modules during bovine pre-implantation development

Pathway analysis revealed essential signaling and metabolic networks in embryonic development. We found more than 100 pathways involved in a sequential order relative to bovine pre-implantation development, most of which were represented in oocytes, major EGA transition (4-cell to 8-cell) and blastocysts (Additional file 18: Table S16). Components of cell cycle, RNA degradation and progesterone-mediated oocyte maturation pathways were highly enriched before the 4-cell stage, while ribosome, spliceosome and proteasome pathways were highly represented after the 8-cell stage. Interestingly, pathways for oxidative phosphorylation, glycolysis, pyruvate metabolism, pentose phosphate and the citrate cycle (TCA cycle), which are critical not only for cell proliferation, but also for maintenance of pluripotency [26], were uniquely found in blastocysts. Additionally, many well-known pathways including MAPK, insulin, ErbB, Wnt, mTOR and TGF-beta signaling were operative in bovine before the 8-cell stage. It is noteworthy that the most prominent changes in biological networks occurred from oocyte to 4-cell stage and blastocyst stage reflecting major functional transitions.

## Discussion

The development of RNA sequencing technologies permits the study of gene regulation at an unprecedented level. Here, we provide the first comprehensive description of gene activities during in vivo bovine embryonic development. Most such studies had been conducted in the mouse [3, 4, 9, 19, 27]. However, mouse data have limited utility in human embryogenesis due to the large differences in gene expression and genome sequences as shown here and in earlier studies. It is therefore important to establish the full expression profile database from an alternative species. To date, all expression profile studies using bovine embryos were either conducted on in vitro embryos and/or using the microarray [8–13, 28]. The few studies employing the RNA-seq technology involved blastocyst stage only [12, 13, 28] except for a recent RNA-seq study using in vitro embryos of multiple stages [10]. None of these reports, however, can be used as the complete “gold standards” for bovine embryo development because in vitro developed embryos have wide-spread gene expression anomalies and the DNA microarray technology limits gene detection to only those printed [8, 11] in addition to variations from hybridization. In this study, we applied the RNA-seq technology and revealed the transcriptomes of bovine in vivo pre-implantation development in a very high-throughput and quantitative manner [14]. For the first time, the bovine matured oocytes and early embryos were shown to transcribe more than half of all bovine genes [22].

The timing of EGA in bovine has long been accepted to be between the 8- and 16-cell stages [6–8]. Using in vivo embryos and microarray containing approximately half of the bovine genome, Kues et al. proposed a new timing: between the 4- to 8-cell stage when the largest number of differentially expressed genes were found [11]. This result was confirmed in our study, also employing in vivo embryos but with all bovine expressed genes, and a more powerful throughput technology, the RNA-seq. However, two prior expression profile studies both using in vitro bovine embryos [8, 10], maintained EGA at the 8- to 16-cell transition. It has been shown through the use of RT-PCR that in vitro vs. in vivo embryos have step-wise differences in mRNA expression [29–31]. The difference in EGA timing among the aforementioned studies therefore further demonstrated that in vitro embryos are not suitable for establishing reference base of early development [32, 33].

Another important contribution of this study was the discovery of patterns of gene expression and their correlation to milestones of embryo development. Four waves of transcriptional changes, between oocyte and 2-cell, between 4- and 8-cell, between 16-cell to early morula, and between compact morula to blastocyst, were each correlated to degradation of maternal RNA, major EGA, compaction and blastulation. These, together with the identification of transient, stage-exclusive gene expression, provide directions of future research in embryogenesis.

Also for the first time, we identified a number of stage-specific modules in bovine pre-implantation development. They not only represent the corresponding stage of embryogenesis, but reveal an interesting progression of core gene networks from cell cycle (oocyte), to regulation of transcription (4-cell), translation (8-cell), stem cell development, maintenance and differentiation (morula), and finally to cell proliferation and protein transport (blastocyst). The identification of these orchestrated functional changes is among the first step to unveil the little-known embryonic programming, and is important in enhancing our ability to improve assisted embryo biotechnologies such as embryo culture conditions. For example, metabolic pathways unique to the bovine blastocysts, such as glycolysis, pyruvate metabolism and the pentose phosphate pathway, were identified. Their presence is compatible with the “Warburg effect” commonly found in cancer cells [34]. In this unique pattern of metabolism, glycolytic end products enter the pentose phosphate pathway instead of the TCA cycle [35]. Such variation from the somatic cells’ metabolism of the TCA cycle [35] not only allows for rapid cell proliferation, but also maintains the pluripotency of the bovine blastocyst [26]. Using this feature of the blastocyst, specific medium that encourages the pentose pathway may be developed to increase the proportion of good embryos in the in vitro production system.

Our cross-species analysis demonstrated that human embryos share more similarity to those of the bovine than the mouse in transcriptomes during early embryonic development. The expression profiles established in this report can therefore serve as a reference base for embryos from assisted technology from both cattle and humans. Interestingly, gene expression profiles unveiled unique developmental programming of embryos in the three species analyzed. At the superficial level differences in stage-specific modules appear to suggest that the bovine embryos progress slower than those of the mouse, but more rapid than those of the human. While this is consistent with the in vivo embryo development between the mouse (3.5 days) [27] and the bovine (8 days) but not between the bovine and the human (5 days) [24]. The inconsistency of gene expression at similar stages of development between humans and bovine suggest that the early embryos employ different pathways to prepare themselves for the upcoming different process of implantation. Moreover, our results showed that the three mammalian species share more maternally deposited genes than EGA-activated genes, concurring with the conclusion from a microarray study using the Bayesian clustering method [9] and again revealing species differences in the programming of embryo development.

The cellular and molecular mechanisms governing mammalian pre-implantation development are still poorly understood. Here we identified a number of hub genes that are critical connectors to other expressed components within each embryonic stage of development in the bovine. Their importance is “validated” by those that have been studied previously. For example, *RALB* is a highly correlated hub gene in oocytes and has key roles in both bovine [36] and Xenopus embryo development [37]. It is also implicated in tumorigenesis and cell proliferation in mice [38]. Similarly, *DNMT3A* is a hub gene in blastocysts. Studies in mice have demonstrated that *DNMT3A* is essential for de novo methylation and embryo development [39, 40]. *DNMT3A* is also likely essential in the bovine blastulation process. The observations that *DNMT3A* is significantly reduced in cloned bovine embryos [41] and that lower pregnancy/calving rates and abnormal development are commonplace in cloned fetuses are “functional validation” of the hub gene status for this important regulator of epigenetic modifications [42].

Most identified hub genes, however, haven’t been studied or annotated, albeit their potential important roles in embryo development. For example, *LOC100137763* and *LOC100849216* are highly correlated, yet un-annotated hub genes identified in oocytes and blastocysts, respectively. The hub genes identified here represent the unprecedented opportunities and insights offered by the RNA-seq technology and bioinformatics. Collectively, our inventories of all hub genes provide a valuable resource for further studies of the molecular mechanisms of pre-implantation development.

Although we had to conduct linear RNA amplification in order to yield sufficient materials from single oocytes/embryos, the highly reproducible protocol employed here has been previously validated [43] and the correlation coefficient after amplification is higher than 0.94 [43]. Readers are cautioned, however, that the in vivo oocytes/embryos used here were generated after superovulation treatment. Although superovulation can affect gene expression of oocytes/embryos [44–47], it is frequently used in both research and production [48] because naturally ovulated/developed oocytes/embryos from single-ovulatory, large animals such as cattle are not very feasible. Nonetheless, the ultimate “gold standards” for gene expression during bovine pre-implantation development can only be established using naturally ovulated/developed oocytes/embryos.

## Conclusion

This study provides comprehensive examinations of gene activities in in vivo bovine oocytes and pre-implantation embryos. Cross-species analysis revealed that bovine pre-implantation transcriptional profiles share more similarity to those of the human than the mice. The data presented here can be used to assess the impact of various assisted reproductive techniques in both bovine and human reproduction.

## Materials and methods

### Ethics statement

Oocytes and embryos were obtained from healthy Holstein cows in the Institute of Animal Science, Xinjiang Academy of Animal Science, Urumqi, Xinjiang, P. R. China. The animal protocol was approved by the Animal Care and Use Committee of Xinjiang Academy of Animal Science (Research license 200815).

### Collection of in vivo matured oocytes and pre-implantation embryos

Multiparous Holstein cows (n = 10) were synchronized and superovulated as described [49, 50]. Artificial insemination using semen from one of three bulls with proven fertility was conducted at 12 and 24 hours post standing heat (Day 0). Donor animals were sacrificed at 30 hours, and 2–4 days after estrus to collect in vivo developed oocytes and embryos at the 2- to 16-cell stages by oviductal flushing. Early morulae, compact morulae and blastocysts were collected by routine non-surgical uterine flushing on Days 5, 6 and 7. All oocytes and embryos were examined and staged under light microscopy. Only morphologically intact embryos meeting the standards of Grade 1 by the International Embryo Transfer Society were included in this study. Embryos were washed twice in D-PBS before frozen and stored individually in RNAlater (Ambion, Grand Island, NY) in liquid nitrogen.

### RNA isolation, linear amplification, library construction and sequencing

Following the reproducible procedures of RNA extraction and linear amplification from our previous study [43], we isolated total RNA from individual oocytes/embryos using TRIzol (Invitrogen, Grand Island, NY) and co-precipitated the RNA with linear acrylamide (Ambion). The quality of the total RNA was examined with the Aglient RNA 6000 Pico kit (Aglient Technologies, Santa Clara, CA) using the Aglient Bioanalyzer 2100. RNA was then amplified twice using the TargetAmp 2-round aminoallyl-aRNA amplification kit 1.0 (Epicentre, Madison, WI) according to the manufacturer’s instructions. 500 ng of amplified RNA (aRNA) were used to construct the sequencing library following the manufacturer’s instructions by SOLiD™ Total RNA-seq Kit (Life Technologies, Grand Island, NY). After the sequencing library was prepared, we used an Agilent 2100 bioanlyzer to analyze the quality of the libraries. The sequencing libraries were then barcoded, multiplexed, and sequenced on a 5500xl Genetic Analyzer at the Center for Applied Genetics and Technology, University of Connecticut. We obtained 430 million sequencing reads with a read length of 75-bp from 16 single oocytes and embryos. The high correlation coefficients between samples of the same development stage demonstrated the reproducibility of the method (Additional file 2: Table S2).

### Mapping, assembly and gene expression analysis

Sequencing adapters were trimmed using Cutadapt (https://code.google.com/p/cutadapt/) and sequencing reads of low quality were pre-filtered by FASTX-Toolkit before mapping (http://hannonlab.cshl.edu/fastx_toolkit/), using the options “fastq_quality_trimmer-Q 33-v-t20-l 30-I”. The quality of reads after filtering was examined using ‘fastQC’ (http://www.bioinformatics.babraham.ac.uk/projects/fastqc/). Filtered reads were mapped to the Btau_4.6.1 assembly using Tophat [51]. Individual mapped reads were fed to Cufflinks [51] to construct transcriptome models. Any novel genes and transcripts that did not fit the supplied gene models (NCBI RefGene) were also assembled. Cuffmerge [51] was used to converge individual transcriptome to produce a master gene model. Genes and transcripts mapped to uncertain chromosomes and contigs were eliminated.

The merged gene model and mapping result BAM files from each RNA-seq library were used to quantify the expression levels of all genes by calculating the number of reads falling into each gene with the Python package, HTSeq [52]. A matrix of Pearson correlation coefficient was created using R, which was in turn used to create the heatmap. Principle component analysis (PCA) was analyzed by using R. Differentially expressed genes between two consecutive developmental stages were identified using default parameters in DESeq [52]. In each comparison, only genes whose sum of expression across all compared samples was greater than the 25th percentile were used. Genes were deemed differentially expressed between subsequent developmental stages if they showed a P-value of less than 0.05 (Negative Binomial Distribution). Expression pattern clusters were generated by the K-means clustering algorithm using R. For gene expression patterns, correlations between pattern indicators and tested genes were calculated. P-values associated with correlations were also calculated and the Bonferroni correction was applied to adjust the P-value for multiple testing. Genes with adjusted P-value of less than 0.05 were considered to have followed the corresponding expression pattern.

### Detection of co-expressed gene modules

The R package for weighted gene co-expression network analysis (WGCNA) [18] was used to detect co-expressed gene modules. A weighted gene co-expression network was first constructed, in which genes were nodes and connected with weighted edges. The connection weights between any pair of two nodes *i* and *j* were computed by  where *corr*(*i*, *j*) is the correlation between the expression levels of nodes *i* and *j* across all stages. The topological overlap matrix *W* which measures the topological similarity of every two genes (nodes) was calculated based on the connection weights as follows:  where  with *d* indexing the nodes that connect to both *i* and *j*, and  with *d* indexing the nodes that connect to the node *i*.

This topological overlap matrix has been shown to produce more biologically meaningful co-expressed gene clusters [17]. We computed the distance matrix *D* by *D*(*i*, *j*) = 1 - *W*(*i*, *j*) A dendrogram of clusters was obtained by applying a hierarchical clustering algorithm [53] on the matrix *D.* The dynamic cutting algorithm reported by Langfelder et al. [54] was used to cut the dendrogram to obtain the clusters of co-expressed genes.

An eigengene was calculated for each cluster as the principal component that explained the largest variance of the data in the cluster. It was a weighted sum of expression profiles of all genes in the cluster where the expression profile of a gene is a vector comprising the values of gene expression at the seven different stages. The eigengene served as the representative of the gene expression profiles in the cluster. Then, clusters whose eigengenes were interrelated with correlation of more than 0.7 were merged. The final clusters of genes were referred to as gene co-expression modules.

### Stage-specific module identification

To detect modules whose eigengene showed high expression levels at a specific stage but low in others, we used a unit vector to indicate each stage. In other words, the entry of this unit vector for the corresponding stage was one, and zero for the others. We then computed the correlations between each stage-indicator vector and the eigengene of each module, which also yielded a P-value associated with each correlation. Smaller P-values corresponded to more significant correlations. If a module received P < 0.05 for the correlation at a particular stage, we labeled the module as specific to that stage.

### Module preservability/reproducibility

We downloaded and mapped genes from two microarray datasets of the bovine [9, 11], and two RNA-seq datasets of the human and mouse [19], as well as our own to the orthologous gene database (http://www.ncbi.nlm.nih.gov/homologene/). After identifying the commonly expressed orthologs, the preservability of a module was measured by the *Z* statistics [20], which characterizes the density and connectivity of genes within a module to those in the validation dataset. The function, module Preservation, in the WGCNA package was used to calculate the *Z* statistics. The categories of preservation were defined as strong if *Z* ≥ 10, weak to moderate if 2 ≤ *Z* < 10, and no evidence of preservation if *Z* < 2, as suggested by an early simulation study [20].

### Cross-species module overlapping analysis

To study if the development of functional modules conserves across species, we compared the gene co-expression modules of the bovine, mouse and human. The same module detection analysis was performed on the human and mouse datasets by Xue et al. [19]. The number of overlapping genes in any two modules each from a different species was counted. Fisher’s exact test was conducted to show whether or not the degree of overlapping was simply due to a random chance, which yielded a P-value reflecting the statistical significance of the overlap.

### Module hub gene identification and validation

The membership of a gene in a module was measured by the correlation between that gene and the eigengene of the module. Genes in a module that are highly correlated with the module eigengene are defined as hub genes for the module. We used all genes with correlation to their module eigengene of greater than 0.9 as the hub genes. To explore the connections among hub genes, we examined the top 200 connections of the top 150 hubgenes for each stage specific module and visualized them in VisANT [55]. To validate the hub genes, we used the raw datasets from two previously published microarray studies in the bovine and one RNA-seq study in the human and mouse. These data were subjected to WGCNA and stage-specific modules and lists of hub genes (kME > 0.9) were generated for each dataset. We then determined the overlap of hub genes from each stage-specific module of the same developmental stage in different datasets.

### Gene ontology analysis

Functional annotation enrichment analysis for Gene Ontology (GO) was conducted by topGo package in R [56]. Database for Annotation, Visualization and Integrated Discovery Bioinformatics Resource [57] was used for pathway analyses. We summarized all similar sub-GO terms and pathways into an overarching term, and P-values are shown for the representative terms.

### Validation of RNA-seq data

Quantitative real-time PCR (qRT-PCR) was performed to validate differential expression of 10 selected genes using embryos at the 4- and 8-cell stages (n = 3). Among these, five genes (*GATA6, GNB2L1, BAD, H2AFZ* and *NANOG*) were up-regulated and five (*GDP9*, *DNMT1*, *ZP2*, *STAT3* and *OOER*) were down-regulated between these two stages. Amplified RNA from individual embryos was reverse transcribed to cDNA by SuperScript III Reverse Transcriptase (Invitrogen) and amplified with specific primers (Additional file 19: Table S17). The qRT-PCR was performed using SYBR Green PCR Master Mix (ABI) and the ABI 7500 Fast instrument. Data were analyzed using the 7500 software version 2.0.2 provided with the instrument. All values were normalized to the internal control, *GAPDH*. The oocytes and embryos from 2-cell to blastocyst stages were pooled and used as the calibrator sample. The relative gene expression values were calculated using the 2^-ΔΔCt^ method. The mean for each stage was determined and compared for an overall fold change.

## Electronic supplementary material

Additional file 1: Table S1: Summary of sequence read alignments to the reference genome. (XLSX 10 KB)

Additional file 2: Table S2: Pearson correlation coefficients of duplicate bovine oocytes and embryos of the same stage. (XLS 26 KB)

Additional file 3: Table S3: Normalized read counts (expression) of genes in bovine oocytes and embryos. (XLS 7 MB)

Additional file 4: Table S4: Differentially expressed genes between the 4- and 8-cell embryos. Spreadsheet 1: all differentially expressed genes; Spreadsheet 2: genes down-regulated; Spreadsheet 3: genes up-regulated; Spreadsheet 4: GO analysis output of down-regulated genes; Spreadsheet 5: GO analysis output of up-regulated genes. (XLS 728 KB)

Additional file 5: Table S5: Differentially expressed genes between compact morulae and blastocysts. Spreadsheet 1: all differentially expressed genes; Spreadsheet 2: genes down-regulated; Spreadsheet 3: genes up-regulated; Spreadsheet 4: GO analysis output of down-regulated genes; Spreadsheet 5: GO analysis output of up-regulated genes. (XLS 308 KB)

Additional file 6: Table S6: Differentially expressed genes between oocytes and the 2-cell embryos. Spreadsheet 1: all differentially expressed genes; Spreadsheet 2: genes down-regulated; Spreadsheet 3: genes up-regulated; Spreadsheet 4: GO analysis output of down-regulated genes; Spreadsheet 5: GO analysis output of up-regulated genes. (XLS 134 KB)

Additional file 7: Table S7: Differentially expressed genes between the 16-cell embryos and early morulae. Spreadsheet 1: all differentially expressed genes; Spreadsheet 2: genes down-regulated; Spreadsheet 3: genes up-regulated; Spreadsheet 4: GO analysis output of down-regulated genes; Spreadsheet 5: GO analysis output of up-regulated genes. (XLS 188 KB)

Additional file 8: Table S8: Differentially expressed genes between the 2- and 4-cell embryos. Spreadsheet 1: all differentially expressed genes; Spreadsheet 2: genes down-regulated; Spreadsheet 3: genes up-regulated; Spreadsheet 4: GO analysis output of down-regulated genes; Spreadsheet 5: GO analysis output of up-regulated genes. (XLS 80 KB)

Additional file 9: Table S9: Differentially expressed genes between the 8- and 16-cell embryos. Spreadsheet 1: all differentially expressed genes; Spreadsheet 2: genes down-regulated; Spreadsheet 3: genes up-regulated; Spreadsheet 4: GO analysis output of down-regulated genes; Spreadsheet 5: GO analysis output of up-regulated genes. (XLS 136 KB)

Additional file 10: Table S10: Differentially expressed genes between the early and compact morulae. Spreadsheet 1: all differentially expressed genes; Spreadsheet 2: genes down-regulated; Spreadsheet 3: genes up-regulated; Spreadsheet 4: GO analysis output of down-regulated genes; Spreadsheet 5: GO analysis output of up-regulated genes. (XLS 114 KB)

Additional file 11: Figure S1: All clusters of expression dynamics during early bovine in vivo embryo development. (TIFF 9 MB)

Additional file 12: Table S11: Distinct Clusters of gene expression patterns in bovine oocytes and embryos. (XLS 91 KB)

Additional file 13: Table S12: Stage-specific/enriched genes in bovine oocytes and pre-implantation embryos. (XLS 43 KB)

Additional file 14: Table S13: Co-expressed genes in stage-specific modules of bovine oocytes and embryos. (XLS 312 KB)

Additional file 15: Table S14: Gene expression overlap between species. Spreadsheet 1: overlapped genes between bovine and human embryos; Spreadsheet 2: overlapped genes between bovine and mouse embryos. (XLS 135 KB)

Additional file 16: Table S15: All hub genes identified in bovine oocytes and pre-implantation embryos (genes in blue are validated in at least one dataset of the human, mouse and bovine). (XLS 86 KB)

Additional file 17: Figure S2: Visualization of representative highly correlated hub genes in bovine oocytes and embryos. (TIFF 10 MB)

Additional file 18: Table S16: Functional pathways in stage-specific modules in bovine oocytes and embryos. (XLS 33 KB)

Additional file 19: Table S17: Primers for real time qRT-PCR. (XLS 21 KB)

## References

[1] Schultz RM (2002). The molecular foundations of the maternal to zygotic transition in the preimplantation embryo. Hum Reprod Update.

[2] Schultz RM (1993). Regulation of zygotic gene activation in the mouse. Bioessays.

[3] Hamatani T, Carter MG, Sharov AA, Ko MS (2004). Dynamics of global gene expression changes during mouse preimplantation development. Dev Cell.

[4] Wang QT, Piotrowska K, Ciemerych MA, Milenkovic L, Scott MP, Davis RW, Zernicka-Goetz M (2004). A genome-wide study of gene activity reveals developmental signaling pathways in the preimplantation mouse embryo. Dev Cell.

[5] Braude P, Bolton V, Moore S (1988). Human gene expression first occurs between the four- and eight-cell stages of preimplantation development. Nature.

[6] Telford NA, Watson AJ, Schultz GA (1990). Transition from maternal to embryonic control in early mammalian development: a comparison of several species. Mol Reprod Dev.

[7] Memili E, First NL (1998). Developmental changes in RNA polymerase II in bovine oocytes, early embryos, and effect of alpha-amanitin on embryo development. Mol Reprod Dev.

[8] Misirlioglu M, Page GP, Sagirkaya H, Kaya A, Parrish JJ, First NL, Memili E (2006). Dynamics of global transcriptome in bovine matured oocytes and preimplantation embryos. Proc Natl Acad Sci U S A.

[9] Xie D, Chen CC, Ptaszek LM, Xiao S, Cao X, Fang F, Ng HH, Lewin HA, Cowan C, Zhong S (2010). Rewirable gene regulatory networks in the preimplantation embryonic development of three mammalian species. Genome Res.

[10] Graf A, Krebs S, Zakhartchenko V, Schwalb B, Blum H, Wolf E (2014). Fine mapping of genome activation in bovine embryos by RNA sequencing. Proc Natl Acad Sci U S A.

[11] Kues WA, Sudheer S, Herrmann D, Carnwath JW, Havlicek V, Besenfelder U, Lehrach H, Adjaye J, Niemann H (2008). Genome-wide expression profiling reveals distinct clusters of transcriptional regulation during bovine preimplantation development in vivo. Proc Natl Acad Sci U S A.

[12] Driver AM, Penagaricano F, Huang W, Ahmad KR, Hackbart KS, Wiltbank MC, Khatib H (2012). RNA-Seq analysis uncovers transcriptomic variations between morphologically similar in vivo- and in vitro-derived bovine blastocysts. BMC Genomics.

[13] Chitwood JL, Rincon G, Kaiser GG, Medrano JF, Ross PJ (2013). RNA-seq analysis of single bovine blastocysts. BMC Genomics.

[14] Wang Z, Gerstein M, Snyder M (2009). RNA-Seq: a revolutionary tool for transcriptomics. Nat Rev Genet.

[15] Ozsolak F, Milos PM (2011). RNA sequencing: advances, challenges and opportunities. Nat Rev Genet.

[16] Eisen MB, Spellman PT, Brown PO, Botstein D (1998). Cluster analysis and display of genome-wide expression patterns. Proc Natl Acad Sci U S A.

[17] Zhang B, Horvath S (2005). A general framework for weighted gene co-expression network analysis. Stat Appl Genet Mol Biol.

[18] Langfelder P, Horvath S (2008). WGCNA: an R package for weighted correlation network analysis. BMC Bioinformatics.

[19] Xue Z, Huang K, Cai C, Cai L, Jiang CY, Feng Y, Liu Z, Zeng Q, Cheng L, Sun YE, Liu JY, Horvath S, Fan G (2013). Genetic programs in human and mouse early embryos revealed by single-cell RNA sequencing. Nature.

[20] Langfelder P, Luo R, Oldham MC, Horvath S (2011). Is my network module preserved and reproducible?. PLoS Comput Biol.

[21] Everts-van der Wind A, Larkin DM, Green CA, Elliott JS, Olmstead CA, Chiu R, Schein JE, Marra MA, Womack JE, Lewin HA (2005). A high-resolution whole-genome cattle-human comparative map reveals details of mammalian chromosome evolution. Proc Natl Acad Sci U S A.

[22] Elsik CG, Tellam RL, Worley KC, Gibbs RA, Muzny DM, Weinstock GM, Adelson DL, Eichler EE, Elnitski L, Guigó R, Hamernik DL, Kappes SM, Lewin HA, Lynn DJ, Nicholas FW, Reymond A, Rijnkels M, Skow LC, Zdobnov EM, Schook L, Womack J, Alioto T, Antonarakis SE, Astashyn A, Chapple CE, Chen HC, Chrast J, Câmara F, Ermolaeva O, Bovine Genome Sequencing and Analysis Consortium (2009). The genome sequence of taurine cattle: a window to ruminant biology and evolution. Science.

[23] Van Soom A, Boerjan ML, Bols PE, Vanroose G, Lein A, Coryn M, de Kruif A (1997). Timing of compaction and inner cell allocation in bovine embryos produced in vivo after superovulation. Biol Reprod.

[24] Niakan KK, Han J, Pedersen RA, Simon C, Pera RA (2012). Human pre-implantation embryo development. Development.

[25] Huang S (2009). Non-genetic heterogeneity of cells in development: more than just noise. Development.

[26] Ito K, Suda T (2014). Metabolic requirements for the maintenance of self-renewing stem cells. Nat Rev Mol Cell Biol.

[27] Li L, Zheng P, Dean J (2010). Maternal control of early mouse development. Development.

[28] Huang W, Khatib H (2010). Comparison of transcriptomic landscapes of bovine embryos using RNA-Seq. BMC Genomics.

[29] Lonergan P, Rizos D, Gutierrez-Adan A, Moreira PM, Pintado B, de la Fuente J, Boland MP (2003). Temporal divergence in the pattern of messenger RNA expression in bovine embryos cultured from the zygote to blastocyst stage in vitro or in vivo. Biol Reprod.

[30] Gutierrez-Adan A, Rizos D, Fair T, Moreira PN, Pintado B, de la Fuente J, Boland MP, Lonergan P (2004). Effect of speed of development on mRNA expression pattern in early bovine embryos cultured in vivo or in vitro. Mol Reprod Dev.

[31] Wrenzycki C, Herrmann D, Lucas-Hahn A, Korsawe K, Lemme E, Niemann H (2005). Messenger RNA expression patterns in bovine embryos derived from in vitro procedures and their implications for development. Reprod Fertil Dev.

[32] Barnes FL, Eyestone WH (1990). Early cleavage and the maternal zygotic transition in bovine embryos. Theriogenology.

[33] Farin PW, Piedrahita JA, Farin CE (2006). Errors in development of fetuses and placentas from in vitro-produced bovine embryos. Theriogenology.

[34] Warburg O (1956). On the origin of cancer cells. Science.

[35] Vander Heiden MG, Cantley LC, Thompson CB (2009). Understanding the Warburg effect: the metabolic requirements of cell proliferation. Science.

[36] Ledgard AM, Lee RS, Peterson AJ (2006). Expression of genes associated with allantois emergence in ovine and bovine conceptuses. Mol Reprod Dev.

[37] Moreau J, Lebreton S, Iouzalen N, Mechali M (1999). Characterization of Xenopus RalB and its involvement in F-actin control during early development. Dev Biol.

[38] Peschard P, McCarthy A, Leblanc-Dominguez V, Yeo M, Guichard S, Stamp G, Marshall CJ (2012). Genetic deletion of RALA and RALB small GTPases reveals redundant functions in development and tumorigenesis. Curr Biol.

[39] Okano M, Bell DW, Haber DA, Li E (1999). DNA methyltransferases Dnmt3a and Dnmt3b are essential for de novo methylation and mammalian development. Cell.

[40] Li E (2002). Chromatin modification and epigenetic reprogramming in mammalian development. Nat Rev Genet.

[41] Beyhan Z, Forsberg EJ, Eilertsen KJ, Kent-First M, First NL (2007). Gene expression in bovine nuclear transfer embryos in relation to donor cell efficiency in producing live offspring. Mol Reprod Dev.

[42] Niemann H, Lucas-Hahn A (2012). Somatic cell nuclear transfer cloning: practical applications and current legislation. Reprod Domest Anim.

[43] Smith SL, Everts RE, Tian XC, Du F, Sung LY, Rodriguez-Zas SL, Jeong BS, Renard JP, Lewin HA, Yang X (2005). Global gene expression profiles reveal significant nuclear reprogramming by the blastocyst stage after cloning. Proc Natl Acad Sci U S A.

[44] Barros CM, Satrapa RA, Castilho AC, Fontes PK, Razza EM, Ereno RL, Nogueira MF (2012). Effect of superstimulatory treatments on the expression of genes related to ovulatory capacity, oocyte competence and embryo development in cattle. Reprod Fertil Dev.

[45] Chu T, Dufort I, Sirard MA (2012). Effect of ovarian stimulation on oocyte gene expression in cattle. Theriogenology.

[46] Mundim TC, Ramos AF, Sartori R, Dode MA, Melo EO, Gomes LF, Rumpf R, Franco MM (2009). Changes in gene expression profiles of bovine embryos produced in vitro, by natural ovulation, or hormonal superstimulation. Genet Mol Res.

[47] Urrego R, Rodriguez-Osorio N, Niemann H (2014). Epigenetic disorders and altered in gene expression after use of Assisted Reproductive Technologies in domestic cattle. Epigenetics.

[48] Mapletoft RJ, Steward KB, Adams GP (2002). Recent advances in the superovulation in cattle. Reprod Nutr Dev.

[49] Hayakawa H, Hirai T, Takimoto A, Ideta A, Aoyagi Y (2009). Superovulation and embryo transfer in Holstein cattle using sexed sperm. Theriogenology.

[50] Lee W, Song K, Lim K, Lee S, Lee B, Jang G (2012). Influence of Factors during Superovulation on Embryo Production in Korean Holstein Cattle. J Vet Med Sci.

[51] Trapnell C, Roberts A, Goff L, Pertea G, Kim D, Kelley DR, Pimentel H, Salzberg SL, Rinn JL, Pachter L (2012). Differential gene and transcript expression analysis of RNA-seq experiments with TopHat and Cufflinks. Nat Protoc.

[52] Anders S, Huber W (2010). Differential expression analysis for sequence count data. Genome Biol.

[53] Hastie T, Tibshirani R, Friedman J (2001). The Elements of Statistical Learning: Data Mining, Inference and Prediction.

[54] Langfelder P, Zhang B, Horvath S (2008). Defining clusters from a hierarchical cluster tree: the Dynamic Tree Cut package for R. Bioinformatics.

[55] Hu Z, Mellor J, Wu J, DeLisi C (2004). VisANT: an online visualization and analysis tool for biological interaction data. BMC Bioinformatics.

[56] Alexa A, Rahnenfuhrer J, Lengauer T (2006). Improved scoring of functional groups from gene expression data by decorrelating GO graph structure. Bioinformatics.

[57] da Huang W, Sherman BT, Lempicki RA (2009). Systematic and integrative analysis of large gene lists using DAVID bioinformatics resources. Nat Protoc.

