# Supplementary figures and images for: Transcriptional profiles of bovine in vivo pre-implantation development
Source: BMC Genomics. 2014 Sep 4;15(1):756. doi: 10.1186/1471-2164-15-756 (PMC4162962; doi:10.1186/1471-2164-15-756)

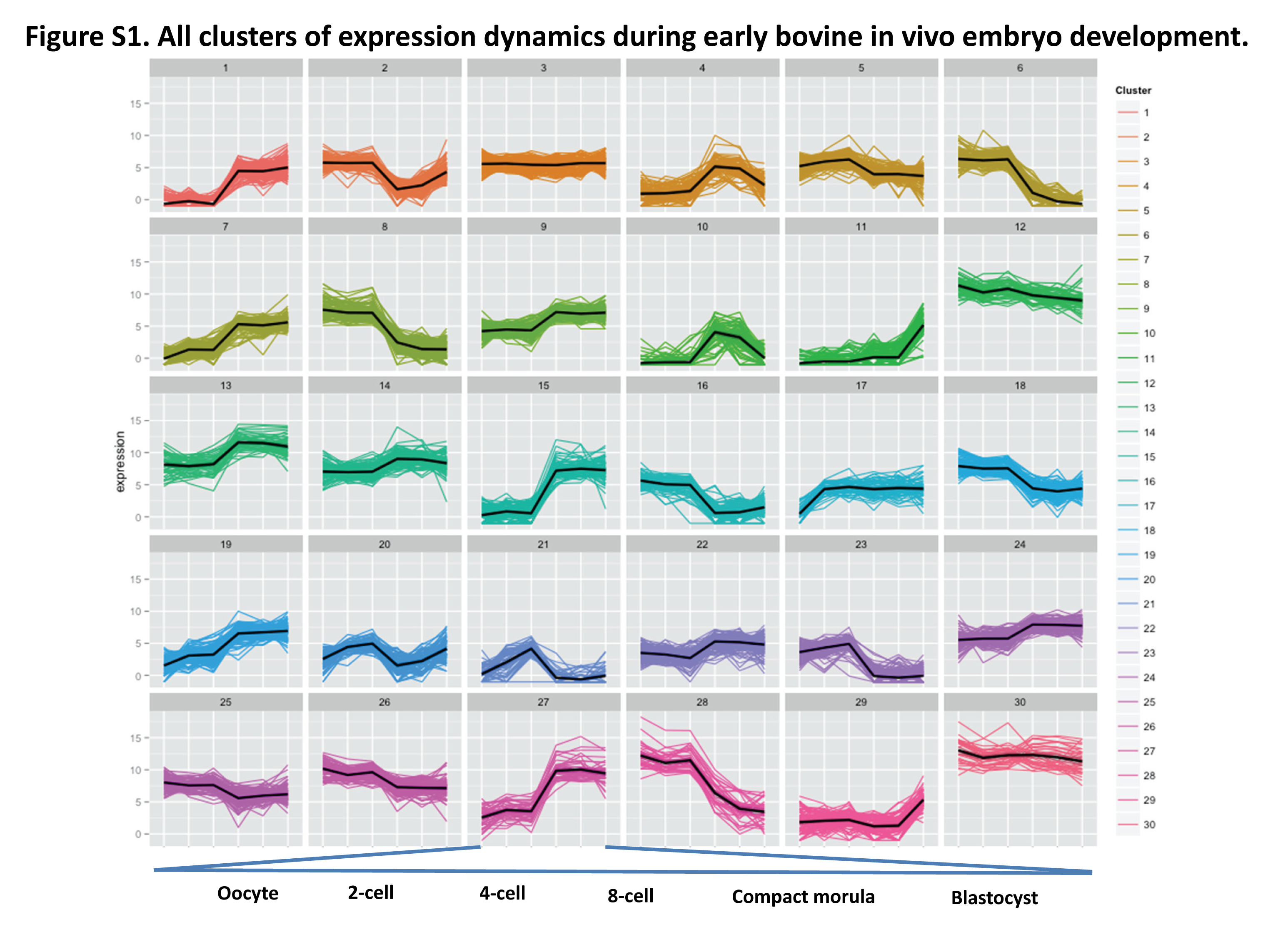

Supplement: Supplementary file 11 — Additional file 11: Figure S1: All clusters of expression dynamics during early bovine in vivo embryo development. (TIFF 9 MB) [file 12864_2014_6433_MOESM11_ESM.tiff]

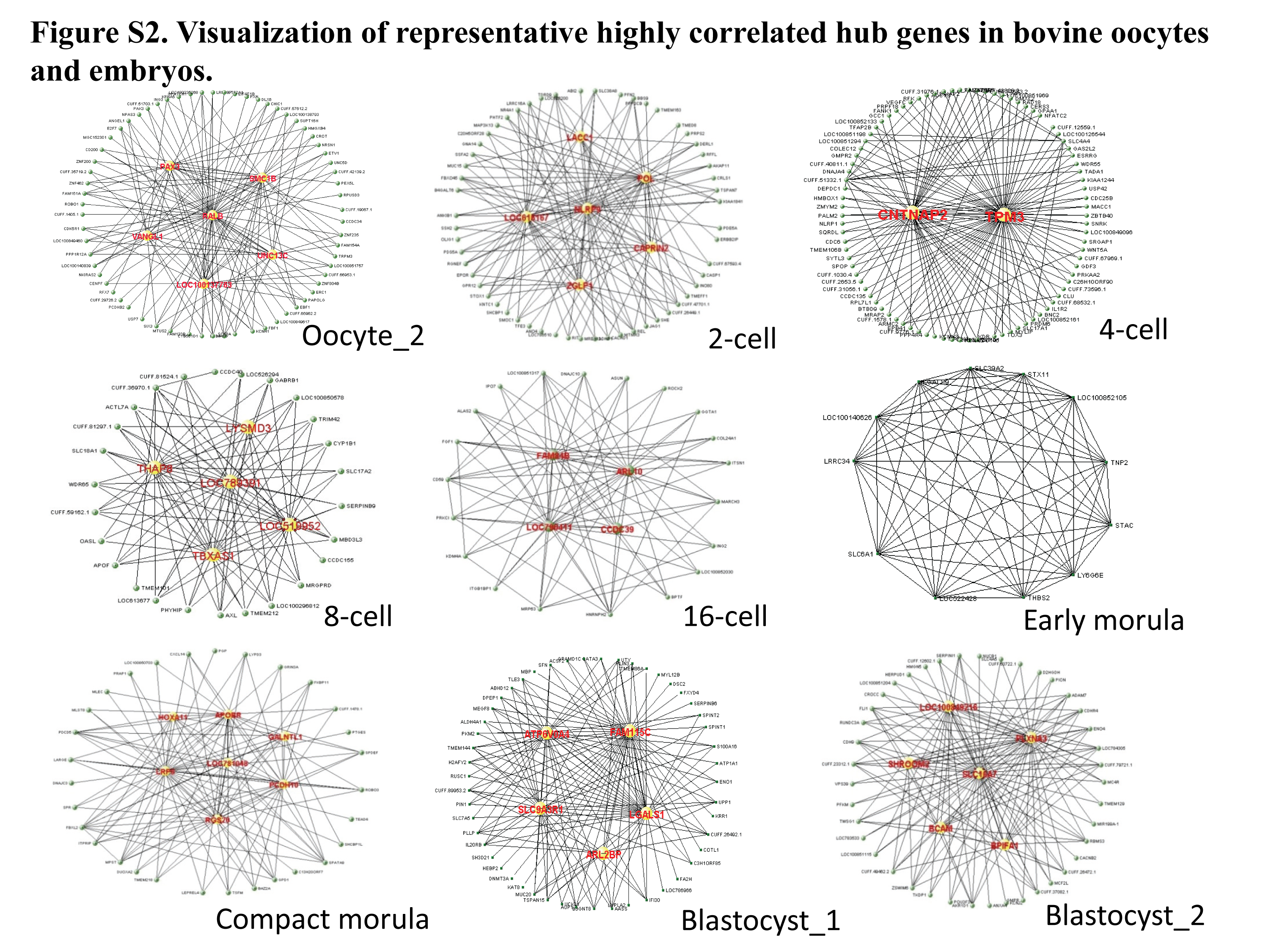

Supplement: Supplementary file 17 — Additional file 17: Figure S2: Visualization of representative highly correlated hub genes in bovine oocytes and embryos. (TIFF 10 MB) [file 12864_2014_6433_MOESM17_ESM.tiff]
